# High-Precision Instance Segmentation Detection of Micrometer-Scale Primary Carbonitrides in Nickel-Based Superalloys for Industrial Applications

**DOI:** 10.3390/ma17194679

**Published:** 2024-09-24

**Authors:** Jie Zhang, Haibin Zheng, Chengwei Zeng, Changlong Gu

**Affiliations:** College of Mechanical Engineering, Zhejiang University of Technology, Hangzhou 310014, China; 18858274591@163.com (H.Z.); 221123020460@zjut.edu.cn (C.G.)

**Keywords:** carbonitride, deep learning, instance segmentation, dispersion

## Abstract

In industrial production, *the* identification and characterization of micron-sized second phases, such as carbonitrides in alloys, hold significant importance for optimizing alloy compositions and processes. However, conventional methods based on threshold segmentation suffer from drawbacks, including low accuracy, inefficiency, and subjectivity. Addressing these limitations, this study introduced a carbonitride instance segmentation model tailored for various nickel-based superalloys. The model enhanced the YOLOv8n network structure by integrating the SPDConv module and the P2 small target detection layer, thereby augmenting feature fusion capability and small target detection performance. Experimental findings demonstrated notable improvements: the mAP50 (Box) value increased from 0.676 to 0.828, and the mAP50 (Mask) value from 0.471 to 0.644 for the enhanced YOLOv8n model. The proposed model for carbonitride detection surpassed traditional threshold segmentation methods, meeting requirements for precise, rapid, and batch-automated detection in industrial settings. Furthermore, to assess the carbonitride distribution homogeneity, a method for quantifying dispersion uniformity was proposed and integrated into a data processing framework for seamless automation from prediction to analysis.

## 1. Introduction

Nickel-based superalloys represent a specialized alloy system designed for applications under elevated temperatures and complex stress conditions. Operating in such environments necessitates exceptional mechanical properties, including high-temperature strength, fatigue and creep resistance, fracture toughness, and microstructure stability [1]. Additionally, these alloys must exhibit surface stability to withstand environmental challenges such as high-temperature oxidation and corrosion. Due to their comprehensive performance in high-temperature operational settings, nickel-based superalloys are extensively utilized in critical hot-end components for engines and gas turbines within the aerospace industry.

The superior performance of nickel-based superalloys at elevated temperatures can be attributed to their intricate chemical composition and sophisticated manufacturing processes. Optimization of their properties predominantly involves alloying strategies, such as the introduction of specific elements like Ti and Nb into the alloys. These elements facilitate the formation of finely dispersed intermetallic compounds such as γ′ phase Ni_3_ (Al, Ti) or γ″ phase Ni_3_ (Nb, Al, Ti), which contribute to precipitation strengthening, solid-solution strengthening, and grain-boundary strengthening. Consequently, these alloys exhibit high strength and exceptional creep resistance in high-temperature environments [2]. However, Nb and Ti also serve as carbide and nitride formers, leading to the precipitation of carbonitride phases during solidification, either in the liquid or solid two-phase region, due to microscopic segregation [3,4]. These are known as primary carbonitrides, and their sizes can be quite large, even reaching tens of micrometers. The enhancement in the mechanical properties of materials by fine precipitates in alloys is unquestionable. However, when the key alloying elements in an alloy fail to effectively form fine or nanoscale-sized precipitates and instead exist in the material as larger, hard particles, these particles are likely to have a negative impact on the mechanical properties of the steel. The detrimental effects of such large-sized hard particles on alloys are comparable to the negative impacts of large, undeformed oxide particles on the fatigue life, impact toughness, and other properties of steel [3,5]. In industrial production, the identification and characterization of micron-sized second phases, such as carbonitrides in alloys, are critical for subsequent composition adjustment and process improvement.

To measure the effect of primary carbonitrides in microstructures, researchers often performed quantitative evaluations of these carbonitrides. The traditional recognition method primarily relied on software such as Image-Pro Plus 6.0 [6]. Import the image into the software and adjust the RGB values to effectively recognize carbonitrides. However, this method mainly had the following disadvantages:

(1) It processed images singly, lacking batch processing capability and necessitating manual intervention, which was time-consuming and labor-intensive.
(2) Images with significant impurities or noise could hinder effective carbonitride recognition despite parameter adjustments, thereby impacting data processing.
(3) The method’s reliance on operator expertise introduced subjectivity into results [7].

Moreover, the high demands on picture quality imposed by Image-Pro Plus 6.0 and similar software further complicate alloy sample imaging and preprocessing in industrial environments, where variability and complexity are prevalent. Consequently, such software may only recognize a subset of high-quality images.

With the development of deep learning technology, the application areas of computer vision become increasingly extensive. Many researchers have started to develop automatic identification models by combining rapid image acquisition techniques and deep learning [8] frameworks. This approach aimed to address the shortcomings of traditional methods and accommodate the unique characteristics of alloy microstructures. Brian et al. [9] investigated and discussed three feature extraction methods: BoW, the VLAD coding, and CNN networks. The SVM algorithm was used to test the performance of each feature extraction method in the task of classification of ultrahigh carbon steel microstructures. Additionally, the clustering and correlation of the microstructures were observed by reducing the high-dimensional space to a two-dimensional space using the t-SNE algorithm. Li et al. [10] utilized the U-Net architecture as a base network model and employed a deep transfer learning approach to identify the γ′ phase in nickel-based high-temperature alloys at 900 °C and 1000 °C, achieving an identification accuracy of 92%. Azimi et al. [11] employed the MVFCNN network structure for the classification and segmentation of martensite, tempered martensite, bainite, and pearlite in mild steel, achieving high accuracy rates. Ghauri et al. [12] utilized the RF algorithm for the segmentation detection of carbides in HP40-Nb stainless steel, achieving notable segmentation accuracies at intergranular and grain boundaries.

Previous generations have conducted a lot of work on the combination of microstructure detection and deep learning model of nickel-based superalloy. For example, Jia et al. [13] used the Unet++ network model to detect the γ′ phase in nickel-based superalloy. They obtained training data through SEM and cut the image size to 512 × 512 to facilitate model training. The Unet++ model is used to segment the γ′ phase, and the mIoU (mean Intersection over Union) value reaches 0.98. That is, the Unet++ network can identify the γ′ phase in nickel-based superalloys well. Senanayake et al. [14] used different methods to identify the γ′ phase and γ″ phase in IN718 alloys, including digital image processing, random forest algorithm (RF), support vector machine (SVM), convolutional neural network (CNN). They used scanned electron phases of IN718 alloy taken at NASA Glenn Research Center as a training dataset. The experimental results show that the use of the convolutional neural network (CNN) can obtain the fastest recognition speed and the most accurate recognition results, and the recognition accuracy rate is 0.95. Previous studies have shown that a deep learning network is the best solution for the identification of microstructure in nickel-based superalloys.

In this study, a microstructure segmentation model was proposed for large-size primary carbonitrides in nickel-based superalloys based on an improved YOLOv8 framework. The improved model for detecting carbonitrides offered a complete alternative to the threshold segmentation method, which satisfied the requirements for high-precision, high-speed, and batch-automated detection in industrial scenarios and was not affected by the quality of the input image. Additionally, the article introduced a method for assessing carbonitride distribution homogeneity, integrating it into a data processing program for automated processing from prediction to analysis. Finally, the YOLOV8-trained model has been able to be deployed in industrial sites, directly applied to on-site production processes, and reduce the operating time of workers. 

## 2. Improved YOLOv8n Instance Segmentation Algorithm

The YOLOv8 algorithm, released in 2023, was one of the most advanced deep-learning models available. It included the following major improvements:

(1) Compared with YOLOv5, YOLOv8 replaced the C3 module in the backbone network with the C2f module. The C2f module integrated the CSP structure and the ELAN [15] concept from YOLOv7. This integration enhanced the feature extraction capability of the YOLOv8 network and reduced computation and model complexity. The SPPF [16] module is retained at the conclusion of the backbone network, facilitating multi-scale feature fusion, thereby enhancing the detection capabilities of the model.
(2) The neck network, similar to YOLOv5, still adopted the PAN-FPN [17,18] feature fusion method. It removed the Conv module in the upsampling stage and replaced the C3 module with the C2f module. These changes maintain the advantages of the YOLOv5 network and improve the detection performance of the model in various scenarios.
(3) For the detection head, YOLOv8 used a decoupled head structure to separate the classification and detection tasks, along with the Anchor-Free [19] algorithm.
(4) The practice of using Mosaic [20] data augmentation during training and turning it off for the last 10 epochs was effective in improving model robustness [21].

Despite these advancements, YOLOv8 encountered challenges in effectively detecting small and densely clustered targets. Previous research has proposed several enhancement methods to address these limitations. For instance, Lou et al. [22] introduced the DC-YOLOv8 algorithm for the detection of small targets captured by cameras. This enhancement to the network’s learning capability was achieved by revising the original downsampling module to an MDC module, swapping out the C2f module for a DC module, and refining the feature fusion process. Li et al. [23] enhanced the Neck layer of the original network by incorporating the BIFPN concept and replacing the C2f module with the Ghostblock module. They also refined the original network’s CIoU with WIoU, applying these improvements to address target detection in UAV aerial imagery. The enhanced model not only reduces the complexity of the model but also the miss rate for small targets. Wang et al. [24] introduced the YOLOv8-QSD network to address the challenge of small target detection in unmanned scenarios. This network incorporated a DBB module in place of the traditional Bottleneck module within the C2f module. It also integrated a BIFPN structure to enhance the original network’s Neck layer. Furthermore, the authors added a novel dynamic detection head, termed DyHead, to the network’s Head layer. With these enhancements, the YOLOv8-QSD network achieved an accuracy of 64.5% on a dataset specifically designed for small target detection.

**Figure 1:**
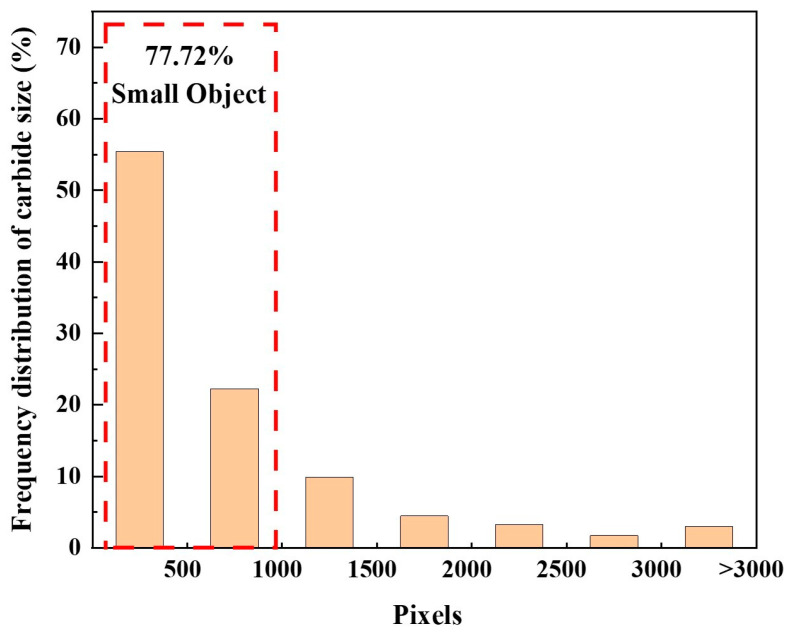
Carbonitride Size Distribution Map.

In industrial applications, the features of alloy carbonitrides were assessed through the examination of microstructures under an optical microscope. In this study, the carbonitride dataset constructed for nickel-based high-temperature alloys was obtained using a metallurgical microscope with image sizes of 2240 × 1524 pixels. The pixel count for individual carbonitrides ranged from approximately 50 to 5000. The MS COCO [25] dataset categorized targets as small if they were below 32 × 32 pixels in size, with the total pixel count for these small targets typically being around or under 1000. In this research, we initially quantified the size distribution of typical carbonitrides in our dataset, as illustrated in Figure 1. The findings revealed that 77.72% of the carbonitrides were classified as small targets, indicating that the majority of carbonitrides examined in this study fall into this category. For ease of calculation, it was assumed that the size of a single carbonitride is 30 × 30, and the number of pixels it occupies is 900. When the training set images were input into the YOLOv8 network, the size of the input image was reduced to 640 × 448, which was 3.5 times smaller compared to the original image, and at this time, the size of the carbonitride was about 9 × 9, which was much smaller than the size of 32 × 32 in the definition of the small target. In summary, the carbonitride dataset for nickel-based superalloys predominantly featured small targets.

In order to illustrate this problem further, we have included more detailed statistics on carbonitrides in the picture. We used the thresholding segmentation method to make more detailed statistics on carbonitrides in the pictures. In order to ensure the accuracy of the statistical results, it is necessary to preprocess the pictures before statistics, such as removing noise and adjusting contrast and brightness. The specific statistical results are shown in Table 1**.** It can be seen from Table 1 that among the 20 carbon nitrides, only the areas (pixels) of 9#, 10#, 12#, and 18# are less than 32 × 32, which is the size definition of small targets in the MS COCO dataset. Thus, it can be clearly seen that most of the targets detected in the carbon-nitride data set are small targets. 

Regarding the average area of a carbonitrides (μm^2^), it can be seen that the area (μm^2^) of a single carbonitrides is from a few square microns to hundreds of square microns, and the average area of these 20 carbon nitrides is about 41.7 μm^2^, calculated in a square, corresponding to a side length of about 6.5 μm.

To enhance the YOLOv8 network’s segmentation capabilities for carbonitrides, this paper mainly improves the YOLOv8n network. We chose YOLOv8n as the basic model of this study for the following reasons: First of all, YOLOv8, as the most advanced deep learning model at present, has a good performance in detection speed and accuracy. Varghese et al. [26] conducted performance tests on YOLOv8 and its previous YOLO series models on COCO data sets. They used Average Precision Across Scales (APAS) and Frames Per Second (FPS) as the evaluation indexes of the models, among which YOLOv8 series models showed the best performance with an APAS score of 52.7 and a FPS score of 150. Compared with the YOLOv7 series model, its indexes are increased by 2.4 and 30, respectively. Secondly, according to the needs of industrial scenarios, the YOLOv8n model is the most suitable choice, considering the training time and calculation amount of the model, as well as the detection accuracy and speed of the model. Finally, the YOLOv8n model is more convenient for model improvement. During the research, we tried to use a larger model and improve it, but its training speed and detection speed were too slow to meet the needs of industrial sites. In addition, the YOLOv8n model also makes it easier to make more adjustments based on subsequent field conditions.

**Figure 2:**
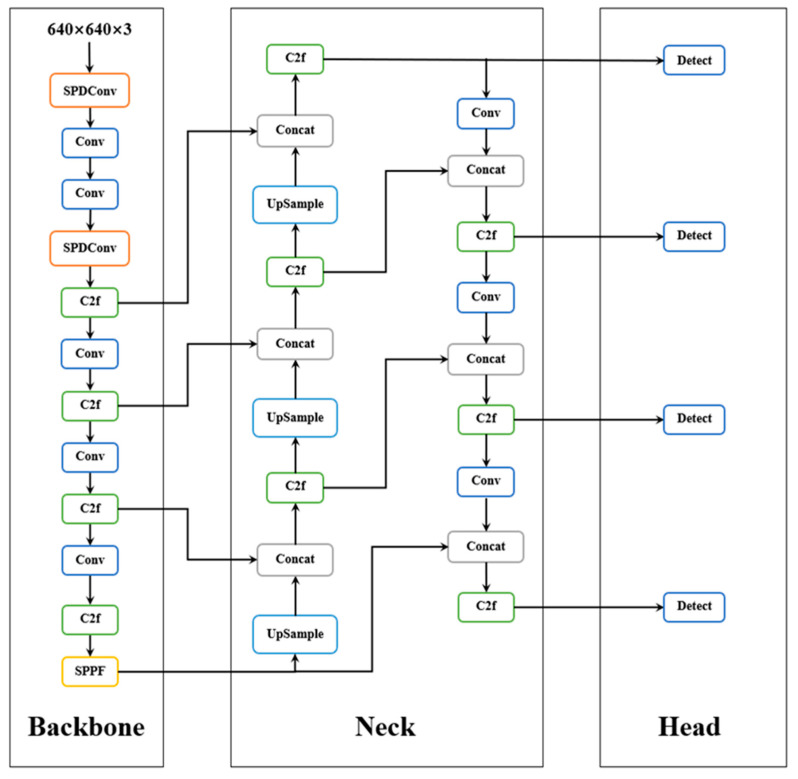
Improved Network Structure [28].

This research proposed several key refinements depicted in Figure 2 [27], and the refinements included:

- Add Space-to-Deep convolution (SPDConv) to the backbone layer.
- Adding a small target detection layer makes the network more focused on small target detection.

### 2.1. Space-to-Deep Convolution (SPDConv)

Space-to-Deep Convolution (SPDConv) was proposed by Sunkara et al. [28], and its purpose was mainly to solve the problem of target detection in the case of small targets and low-resolution images. In general detection scenarios, images possess high resolution, and the targets are of moderate size, meaning that much of the images contain redundant information. These excess data could be effectively filtered through convolutional operations, residual connections, and pooling layers, allowing the model to discern and learn the essential features of the targets. However, in low-resolution images with small targets, the presence of redundant information was minimal. Continuous downsampling by the model could result in the loss of critical feature information for these targets, potentially rendering them undetectable.

SPDConv primarily comprised a space-to-depth layer and a non-strided convolutional layer. It began by taking an input feature map of size (S, S, C1). The space-to-depth layer then rearranged the input X into multiple sub-feature maps, with the transformation calculated as follows:

(1) $$f_{\left(0,0\right)}=X[0:S:scale,0:S:scale],\;f_{\left(1,0\right)}=X[1:S:scale,0:S:scale],\;\vdots$$

(2) $$f_{\left(scale-1,0\right)}=X[scale-1:S:scale,scale-1:S:scale];$$

(3) $$f_{\left(0,1\right)}=X[0:S:scale,1:S:scale],\;f_{\left(1.1\right)}=X[1:S:scale,1:S:scale],\;\vdots$$

(4) $$f_{\left(scale-1,1\right)}=X[scale-1:S:scale,scale-1:S:scale];$$

(5) $$f_{\left(0,scale-1\right)}=X[0:S:scale,scale-1:S:scale],$$

(6) $$f_{\left(1,scale-1\right)}=X[1:S:scale,scale-1:S:scale],\;\vdots$$

(7) $$f_{\left(scale-1,scale-1\right)}=X[scale-1:S:scale,scale-1:S:scale]$$

**Figure 3:**
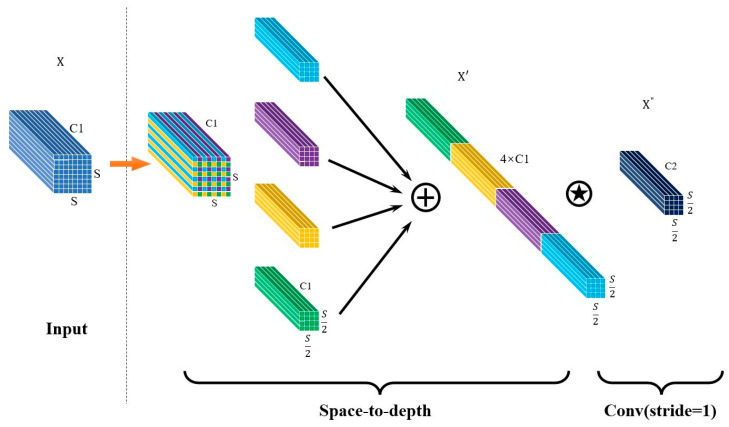
SPDConv Structure Diagram.

Then, several sub-feature maps were concatenated along the channel direction to obtain $X^{\prime }$ with dimensions (s/scale, s/scale, scale^2^C1). If the scale was taken as 2, the calculation process was illustrated in Figure 3. In Figure 3, the plus sign indicates that all sub-feature graphs are spliced according to the channel direction, and the five-pointed star indicates that the convolution calculation with step size 1 is performed on the spliced feature graphs.The dimensions of $X^{\prime }$ would be (s/2, s/2, 4×C1 ). Subsequently, a non-strided convolution was applied to transform $X^{\prime }$ into a new feature map $X^{\prime \prime }$ with dimensions (s/2, s/2, C2), where $C2<{scale}^{2}C1$. The use of non-strided convolution here primarily aimed to retain all relevant information. While transformations from X to $X^{\prime \prime }$ were possible with strides greater than 1, they might result in the loss of some features.

In order to further illustrate the role of the SPDConv module for small target detection, we use a concrete example. Suppose the input dimension is (640, 640, 3); after passing through the space-to-depth layer, its dimension becomes (320, 320, 12), and then the number of channels is modified by the convolution module with step 1. After such operation, the feature information of small and medium-sized objects in the picture can be well preserved. That is, the SPDConv module rearranges the spatial dimension information to the depth dimension, thereby avoiding the information loss caused by traditional step volume. If the convolution check with the size of 3 × 3 is used for the convolution operation of the input dimension, although the feature graph size of 320 × 320 can be obtained, the feature information of the small target will be lost during the convolution process, resulting in the failure of the model to recognize the small target, and the subsequent data processing will be seriously affected.

### 2.2. Small Target Detection Layer

The original YOLOv8n network primarily operated on feature maps sized at 80 × 80, 40 × 40, and 20 × 20. However, during downsampling, features representing carbonitrides might diminish to a few pixels or vanish entirely. To address this issue, Zhai et al. [29] investigated and enhanced the Neck and Head layers of the YOLOv8n network. The 160 × 160 scale feature maps were introduced in the Neck layer to enhance feature fusion, emphasizing that this larger scale better-preserved feature information for small targets and enhanced overall detection accuracy. Concurrently, a corresponding detection head in the Head layer was integrated, utilizing four detection heads collectively to optimize model performance.

**Figure 4:**
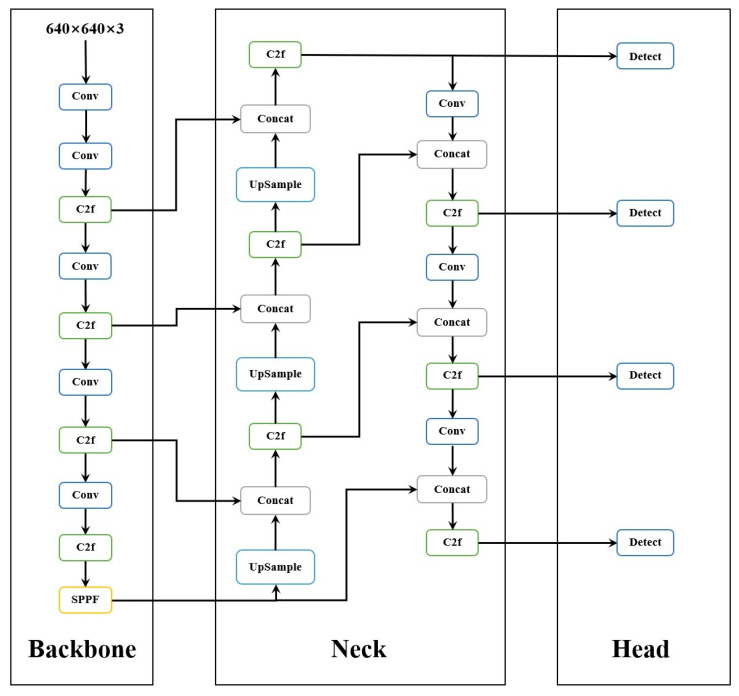
Small target detection layer.

In order to further explain the role of the P2 small target layer, we use the structure of the P2 small target layer to illustrate. Its structure diagram is shown in Figure 4. As can be seen from the structure diagram, a P2 small target layer is added to the network structure, and four detection heads are used for multi-scale detection. Among them, the feature map size of the P2 small target layer is 160 × 160, which has a higher resolution and can give a finer representation of small targets.

## 3. Experiments

### 3.1. Image Acquisition

In this experiment, metallographic specimens sampled from nickel-based superalloy bars of different dimensions were selected. The specimens were prepared by grinding and polishing without etching. Images were captured using an optical microscope (OM, Olympus, Tokyo, Japan) at 200× magnification, resulting in a dataset where each photograph had a resolution of 2240 × 1524 pixels. This dataset included two types of compounds: TiN and NbC.

### 3.2. Data Annotation

**Figure 5:**
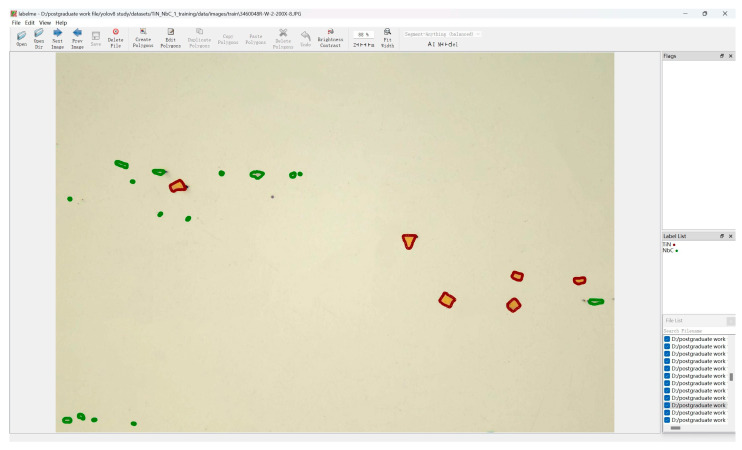
Labelme Labeling Software Interface.

All images within the dataset underwent preprocessing and were subsequently annotated for carbonitrides using the Labelme v1.8.1 software. During annotation, the delineation precisely matched the contour of the carbonitride. The detailed annotation view and software interface are shown in Figure 5, where the green contour indicates NbC and the red contour indicates TiN. After completion of the annotation process, a JSON file was generated. This file primarily contained the positional information of the carbonitride contours and the category names of the carbonitrides. Subsequently, the JSON file was converted into a TXT file to enable the improved YOLOv8 network to recognize the annotation information, ensuring the normal progression of subsequent training.

### 3.3. Data Amplification

To ensure the effectiveness and enhance the generalization of the model, the original dataset was augmented. Image enhancement techniques were employed, including mirroring, Gaussian noise addition, brightness adjustment, and random point overlay. The augmented dataset comprised 1530 images, evenly split into training, validation, and test sets at a ratio of 8:1:1.

### 3.4. Model Training

The hyperparameters used in the experiment are shown in Table 2. Upon completion of training, a model file named ‘best.pt’ was generated, which was utilized for subsequent carbonitride prediction tasks.

## 4. Results and Discussion

### 4.1. Experimental Environment

For this experiment, the compiler used was Python 3.8, with PyTorch version 2.0.0 and CUDA version 11.8. On the hardware front, the CPU was an Intel (R) Xeon (R) Platinum 8474C, and the graphics card was an RTX 4090D with 24 GB of memory.

### 4.2. Model Performance Evaluation

To assess model performance, the following key metrics were selected for evaluation: the confusion matrix, Precision (P), Recall (R), and mean Average Precision (mAP). 

The confusion matrix is a tabular form used to evaluate the performance of a classification model. It serves as a visualization tool, primarily for comparing classification outcomes with actual measured values, and it can display the accuracy of classification results within the matrix. Taking binary classification as an example, the confusion matrix is shown in Table 3. In it, TP (True Positive) indicates that the sample’s actual value category is the positive class, and the model identification result is also the positive class. FN (False Negative) indicates that the sample’s actual category is the positive class, but the model identification result is the negative class. FP (False Positive) indicates that the sample’s actual category is the negative class, but the model identifies it as the positive class. TN (True Negative) indicates that the sample’s actual value is the negative class, and the model also identifies it as the negative class. Subsequent advanced evaluation metrics are also calculated based on these four parameters of the confusion matrix.

Precision (P) refers to the proportion of data correctly predicted as the positive class among all data predicted as positive by the model. The calculation formula is:

(8) $$P=\frac{TP}{\left(TP+FP\right)}$$

Recall (R) refers to the proportion of the actual positive instances in the sample that are correctly identified by the model. The calculation formula is:

(9) $$R=\frac{TP}{\left(TP+FN\right)}$$

Mean Average Precision (mAP) is an important metric for evaluating model performance. A higher mAP value indicates better model performance. Before calculating mAP, you need to calculate the Average Precision (AP) for each class first. The calculation formula is:

(10) AP=∫P(R)dR

In the formula, *P*(*R*) refers to the function curve of Precision (P)–Recall (R) for a single class. From this, the value of mAP can be calculated, with the calculation formula being:

(11) $$mAP=\frac{1}{n}\sum_{i}^{n}{AP}_{i}$$

In the formula, *n* represents the total number of classes. In this experiment, *n* = 2.

### 4.3. Comparison of SPDConv Module Improvement Effects

**Figure 6:**
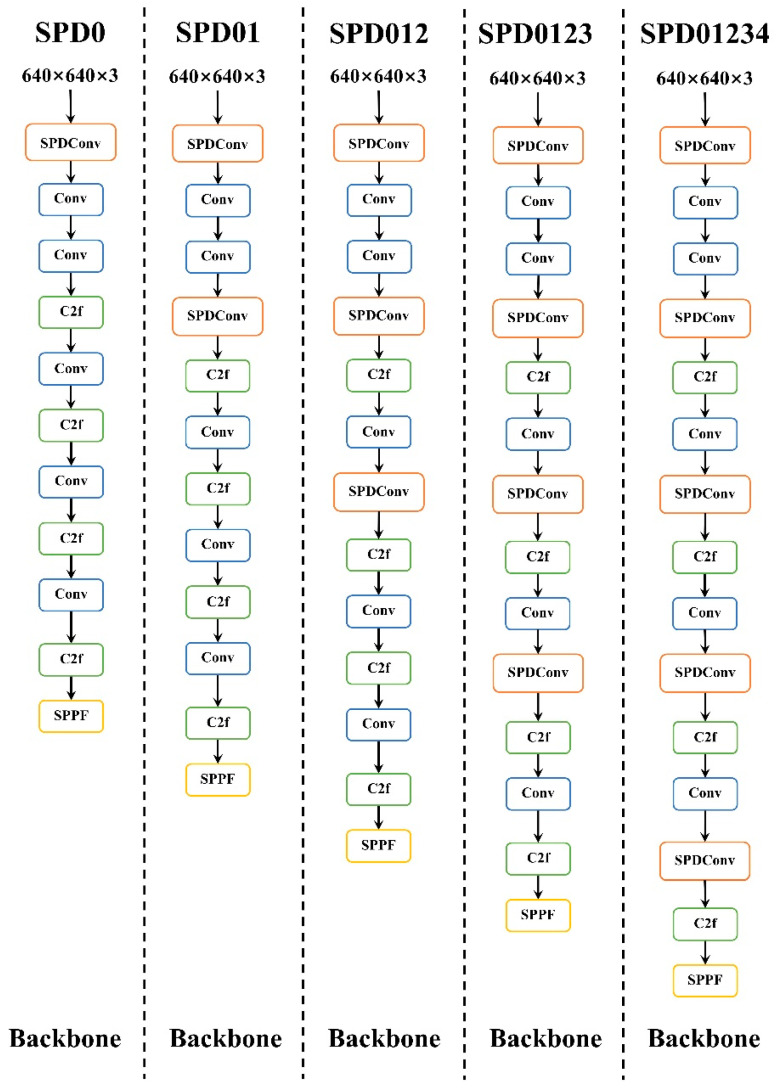
SPDConv Module Different Add Locations.

To verify the adaptability of the SPDConv module to the overall model, comparative experiments were conducted by adding the SPDConv module to different positions within the model. The specific locations of addition are shown in Figure 6. SPD0 indicates that the SPDConv module is added at the 0th layer of the backbone. SPD01 indicates that the SPDConv module is added at the 0th and 3rd layers of the backbone, a method of addition that is consistent with the improvement approach in this research. SPD012 indicates that the SPDConv module is added at the 0th, 3rd, and 6th layers of the backbone. SPD0123 indicates that the SPDConv module is added at the 0th, 3rd, 6th, and 9th layers of the backbone. SPD01234 indicates that the SPDConv modules are added at the 0th, 3rd, 6th, 9th, and 12th layers of the backbone. The comparative experimental results for TiN and NbC are shown in Table 4 and Table 5, respectively. The main evaluation metrics selected are Precision (P), Recall (R), and mAP50.

Data from Table 4 and Table 5 show that, considering detection and segmentation accuracy alongside computational load, the SPD01 structural improvement is the most effective. For TiN, the mAP50 values for the detection box and mask are 0.808 and 0.576, increasing by 4.6% and 5.4% from the unimproved model. For NbC, these values are 0.622 and 0.439, with increases of 3.1% and 1.8%. Consequently, this research selected the SPD01 module for improvement.

### 4.4. Heatmap Visualization and Analysis

**Figure 7:**
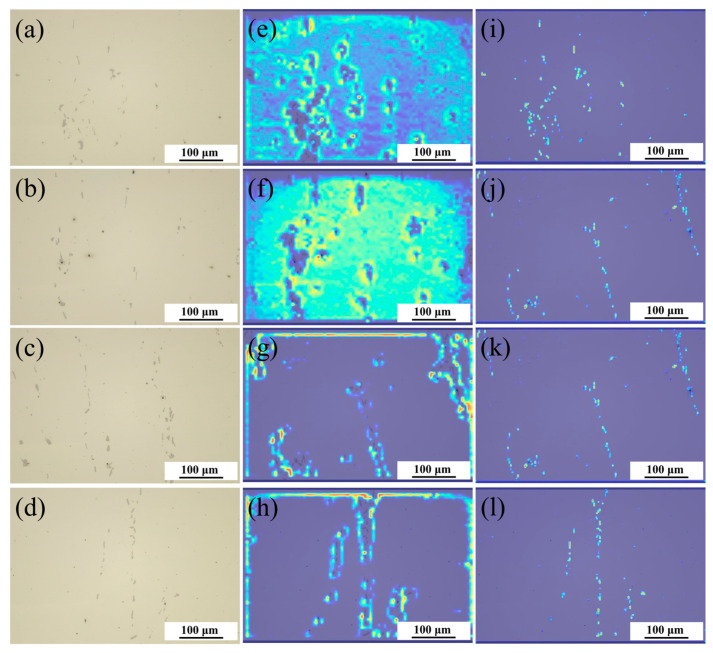
Visualization of Heatmap Results. (**a**–**d**): Original Image; (**e**–**h**): Unimproved model; (**i**–**l**): The Model with SPD01 Layer added.

In order to further clarify the mechanism of the SPD01 module, this research used the GradCAM [30] method to visualize the attention region of the model by generating a heat map, and the specific effect is shown in Figure 7, where the closer the color is to red, the more attention the model pays to the region.

Figure 7 illustrates that the unimproved model attention is focused on the background and edges of the image, or it only partially captures the carbonitrides and sometimes fails to focus on them at all. After adding SPD01, the model’s attention to carbonitrides was significantly improved, its attention accuracy increased substantially, and it was also able to distinguish between impurities and carbonitrides. In summary, the SPD01 module significantly improved the model recognition rate and model performance for carbonitrides by increasing the model attention to small targets.

### 4.5. Ablation Experiment

To verify the overall effect of the improved model, YOLOv8n was used as the baseline model, and ablation experiments were conducted on each improved module. The effectiveness of the model improvements was determined by comparing evaluation metrics such as Precision, Recall, and mAP50 values.

The ablation experiment results for TiN and NbC are detailed in Table 6 and Table 7, respectively. For clarity, the small target detection layer is referred to as “small”. In Table 6 and Table 7, “√” indicates that the module was added to the model and “×” indicates that the module was not added to the model. As indicated in Table 6, the addition of only the SPDConv module to TiN results in a 4.6% and 5.4% increase in mAP50 values for the detection box and mask, respectively, compared to the baseline model. When the small layer is added alone, the mAP50 values for the detection box and mask rise by 9.3% and 15.9%, respectively. With both modules incorporated, the mAP50 values for the detection box and mask see an increase of 11.5% and 20.8%, respectively. Table 7 reveals that for NbC, the addition of SPDConv alone leads to a 3.1% and 1.8% increase in mAP50 values for the detection box and mask, respectively. When the “small” layer is added alone, the mAP50 values improve by 15.4% for the detection box and 13.5% for the mask. Upon adding both modules, the mAP50 values for the detection box and mask increased by 18.9% and 13.6%, respectively. These findings underscore that the enhanced network significantly bolstered model performance and enhanced the precision of carbonitride detection and segmentation.

### 4.6. Model Prediction Effect Comparison

**Figure 8:**
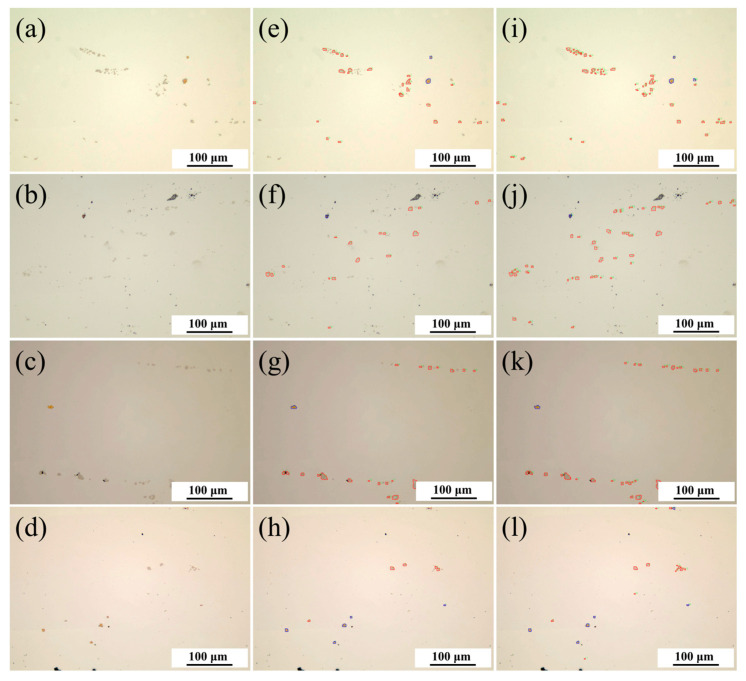
Comparison of The Effects Before and After Prediction. (**a**–**d**): Original Image; (**e**–**h**): Unimproved Model; (**i**–**l**): Improved Model.

After training the model, the batch prediction was programmed using PyCharm 2023.2.1 software. The parameters used for prediction are shown in Table 8. The prediction results before and after the improvement under the same parameter conditions are shown in Figure 8, where the blue contour represents TiN, and the red contour represents NbC. It can be observed that the unimproved model has a high false-negative rate for dense, small-sized carbonitrides, with a large number of NbCs not being correctly identified; the improved model can more easily detect them, reducing the false-negative rate, and also has good discrimination ability for impurities. Overall, the improved model was more effective for detecting carbonitrides in high-temperature alloys.

### 4.7. Data Processing

To analyze the impact of carbonitrides on nickel-based high-temperature alloys, the data of the masked area were processed at the same time when using the model prediction. The main metrics selected for calculation include the number of carbonitrides, centroid coordinates, area of the region, area fraction, and dispersion.

### 4.8. Calculation of Carbonitride Dispersity

During the process improvement of nickel-based superalloys, the uniformity of the distribution of carbonitrides was found to have a critical impact on the differences in transverse and longitudinal properties of the nickel-based superalloys. It was possible to accurately quantify the average size, number, and other characteristics of carbonitride particles, but there was no suitable method for the indicator of the uniformity of the distribution of carbonitride particles. After searching through the literature, no reliable research basis could be found.

**Figure 9:**
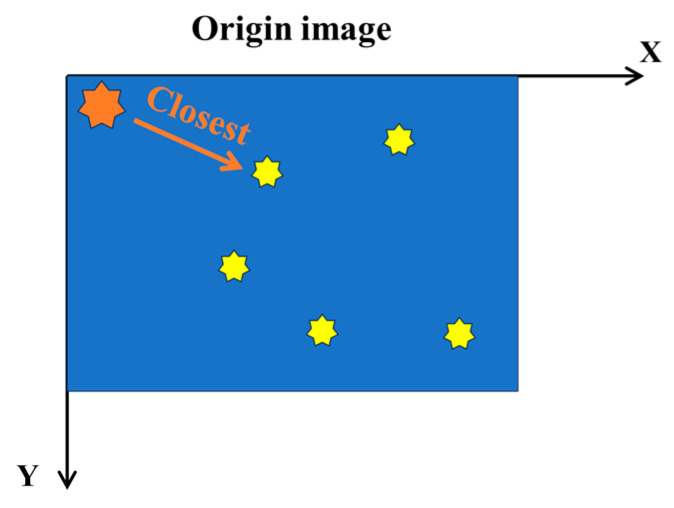
Schematic of Carbonitride Dispersion Statistics.

The objective of this research was to quantitatively evaluate the uniformity of the distribution of carbonitride particles. This question could be generalized into a standard computational measure: the quantification of particle dispersion. Specifically, that is calculating the standard deviation of the distances between each mass point and other mass points, as shown in Figure 9. Ideally, if the distribution of mass points was completely uniform, the standard deviation of the distance between each mass point and its nearest neighboring mass point should be zero. At this point, the dispersion of carbonitride particles was zero, indicating that the distribution of carbonitride particles was absolutely uniform.

**Figure 10:**
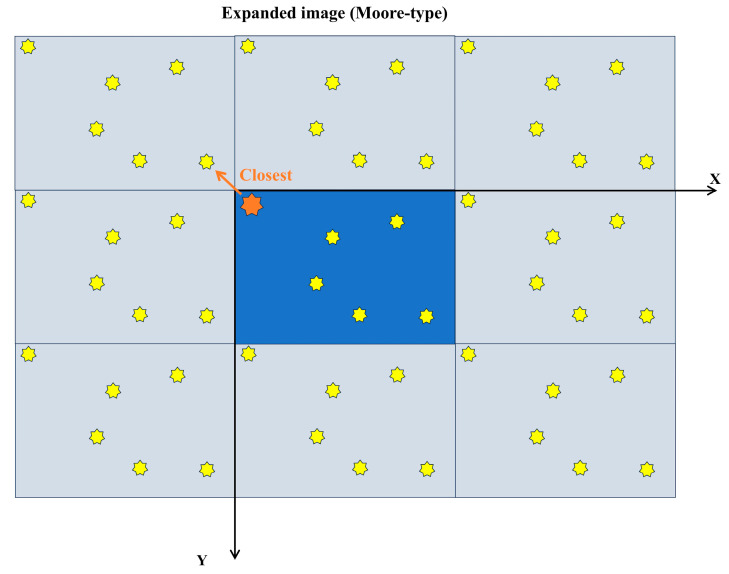
Schematic of the expanded image (Moore-type neighborhood).

Considering the limited area that could be sampled in each image, the nearest particles of carbonitrides near the edge of the image might not be present within the same image. This could lead to an overestimation of the measured dispersion. To address this issue, this study adopted the Moore neighborhood type from cellular automata, duplicating the original image eight times and translating it to form eight adjacent neighborhoods around the original image (1 × 1), creating a new image array (3 × 3). By calculating the distance from each carbonitride particle in the central image (1 × 1) to the nearest carbonitride particle in the entire new image (3 × 3), we could mitigate the bias in the dispersion measurement compared to the actual value. This approach is illustrated in Figure 10.

In Figure 9 and Figure 10, both the yellow and orange heptagon stars represent carbonitrides, the blue background represents the original image, and the white background represents the reproduced image.

However, simply calculating the standard deviation of the distance between each particle and the nearest particle could still lead to misjudgment. When the distribution of particles was extremely uneven, such as when large-sized primary carbonitrides were just slightly broken and clustered together, it was observed that the calculated dispersion was also relatively low. However, under actual conditions, the distribution of carbonitrides in this situation was not uniform. This was an inevitable problem encountered when calculating the nearest neighbor distance because it only considered the standard deviation of the shortest distance between particles, ignoring the distance between particles and edges or corners. Considering that the number of actual carbonitride particles was not sufficiently large, the array composed of the shortest distances was supplemented with the nearest distance from the particle group to the four corners of the rectangle. At this time, a new dispersion could be calculated.

**Figure 11:**
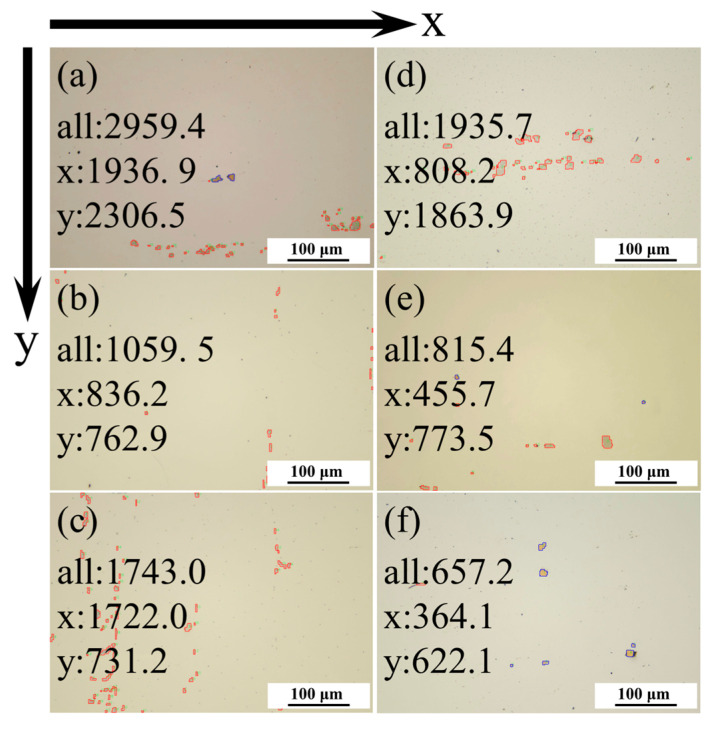
Dispersion calculation results (**a**): 1-1# (**b**) 1-2# (**c**) 1-3# (**d**) 1-4# (**e**) 1-5# (**f**) 1-6#.

The dispersion calculation program is embedded into the carbonitride recognition program, and the final data processing results are shown in Table 9. The corresponding image effect diagrams are shown in Figure 11.

### 4.9. Advantages in Industrial Scenarios

The complexity and uniqueness of engineering problems result in a lack of common data sets in our field, making direct comparisons with experimental results from other authors difficult. At the same time, previous methods used in this field are based on threshold segmentation, such as Image-Pro Plus 6.0 and other software for segmentation and statistics. For example, Chen et al. [31] analyzed the effects of dual melting (VIM + VAR) and triple melting (VIM + ESR + VAR) technologies on the properties of superalloy GH4738 by means of XRD, SEM, EDS, and Image-Pro Plus software. The Image-Pro Plus software is mainly used to count the number and size of inclusions in the alloy. Yang et al. [32] used characterization methods such as SEM and Image-Pro Plus software to observe and quantify the microstructure in dual-phase Ti-6AI-4V alloy to analyze its mechanical properties. The Image-Pro Plus software is mainly used to calculate the number and size of grains in the alloy. Specific statistical accuracy is not given in the literature.

In order to verify the validity and universality of our method, we will conduct a comparative analysis from two aspects:

(1) More extensive literature research. Related research in other fields has shown that approaches based on computer vision and deep learning have significant advantages over traditional approaches based on threshold segmentation. For example, Wang et al. [33] used ResNet network and Image-Pro Plus 6.0 software to count the number of nuclei in the process of cell proliferation, in which the average accuracy of Image-Pro Plus 6.0 software was 67.2%, and the average accuracy of ResNet network recognition was 90%. Zhu et al. [34] used the improved FCN network and Image-Pro Plus software to segment the eutectic silicon in Al-Si alloy, calculate the parameters of its microstructure characteristics, and compare with the theoretical results. The results obtained by the improved FCN network segmentation were closer to the theoretical values.

(2) Data statistics. In the actual industrial application environment, the improved model in this study had significant advantages in both accuracy and speed. To illustrate this, this research compared the identification results of Image-Pro Plus 6.0 software based on threshold segmentation with the instance segmentation results of the improved model and used manual statistical data as a benchmark to measure the accuracy of the two detection methods by counting the number of carbonitrides. In the Image-Pro Plus 6.0 software, the set values for R, G, and B are 200, 190, and 185, respectively, and the minimum segmentation recognition area was set to 10 to filter out the noise in the image. The specific comparison results are shown in Table 10, from which it can be seen that the number of identifications by the improved model is closer to the manual statistical results, with the recognition error between zero and five. Although the minimum recognition area was set in the Image-Pro Plus 6.0 software, due to the impact of image quality, there were still many noise points and impurities recognized. The identification results of the Image-Pro Plus 6.0 software were about three-to-seven times the manual statistical results, some of which were affected by noise and impurities, significantly affecting the subsequent data processing results. In summary, the improved model had superior performance and far exceeded the Image-Pro Plus 6.0 software in detection speed and accuracy. At the same time, the model could be used as a pre-trained weight file for automatic annotation software after processing for automatic segmentation of micron-level carbonitrides in other steel grades, significantly accelerating the detection and instance segmentation tasks of carbonitrides in other steel grades.

In order to further illustrate the effectiveness of the improved model, based on the actual data of the industrial field, a detailed statistical analysis is carried out. With the manual statistical data as a reference, the recognition effect of deep learning and threshold segmentation method is compared by calculating Precision and Recall. The calculation results of the two methods are shown in Table 11. From Table 11, it is obvious that the average value of the improved YOLOv8 model in terms of Precision and Recall exceeds 90%. Although the Recall of the recognition results of Image-Pro Plus 6.0 is high due to the impact of the shooting environment of industrial site pictures and the lack of preprocessing of pictures, it can recognize many impurities—that is, the Precision is low. In summary, the recognition accuracy of the improved YOLOv8 model is far higher than that of the recognition based on the threshold segmentation method.

### 4.10. Practical Challenges and Industrial Relevance of Model Implementation

The ultimate goal of this research is to build a system in which the AI model can iterate itself, which also brings challenges to our work. Encouragingly, the model has significant versatility and growth. After industrial mass production has shown the characteristics of accuracy, speed, standard, and low labor cost, the model has been rapidly applied to the carbonitrides quantitative statistics of other grades of the industrial production process. In the future, we need to obtain more microstructure photos of steel varieties to continuously improve the performance of the model and build the above system.

In the subsequent research, more improvement methods will be applied, such as adding CBAM and EMA attention mechanisms, trying more loss functions, changing the feature extraction strategy of the backbone network, modifying the feature fusion mechanism of the neck network, etc. Through these methods, the performance of the model is again improved to meet the needs of the industrial field. Beyond that, existing improved models can be leveraged to process new data and added to datasets that can be used to train more general models. Implement the iterative update of the AI model.

## 5. Conclusions

(1) The structural improvement of SPD01 mapped spatial dimension information to depth dimension, effectively addressing the problem of feature information loss. Heatmap visualization indicated that the SPD01 module enhanced model attention on carbonitrides, with substantial accuracy gains. The mAP50 values for TiN and NbC masks improved by 5.4% and 1.8%, respectively.

(2) By incorporating a specialized P2 layer, the model was endowed with a more refined recognition capability for small targets, thereby significantly enhancing the segmentation accuracy for these targets. The mAP50 values for TiN and NbC masks improved by 15.9% and 13.5%, respectively.

(3) Compared to the original network, integrating SPD01 and the P2 layer further enhanced model performance, increasing precision in carbonitride detection and segmentation while reducing missed detection rates. The mAP50 values for TiN and NbC masks improved by 20.8% and 13.6%, respectively.

(4) A method and program for calculating the dispersity of carbonitrides developed and embedded into the carbonitride recognition program, enabling batch detection and data processing. The current model was capable of performing high-precision instance segmentation detection tasks for primary carbonitrides in industrial scenarios.

## Figures and Tables

**Table 1:** Carbonitrides statistics.

Obj.	Type	Area (Pixels)	Area (μm^2^)	Center-X	Center-Y
1#	TiN	604	34.8	737.1	463.5
2#	TiN	405	23.3	1943.0	584.0
3#	TiN	590	34.0	803.5	717.3
4#	NbC	289	16.6	314.1	895.9
5#	NbC	81	4.7	1406.2	936.9
6#	NbC	456	26.3	315.0	951.9
7#	NbC	122	7.0	714.4	969.0
8#	NbC	766	44.1	1075.6	972.7
9#	NbC	2129	122.6	1369.2	999.0
10#	NbC	2344	135.0	1162.1	1000.5
11#	NbC	625	36.0	1098.9	1007.0
12#	NbC	2043	117.7	993.8	1021.9
13#	NbC	54	3.1	967.6	1004.8
14#	NbC	858	49.4	910.4	1020.7
15#	NbC	51	2.9	952.5	1032.4
16#	NbC	122	7.0	1219.1	1041.7
17#	NbC	875	50.4	953.7	1065.4
18#	NbC	1053	60.7	1476.4	1121.9
19#	NbC	584	33.6	1246.6	1121.2
20#	NbC	416	24.0	1170.0	1127.0

**Table 2:** Hyperparameter Settings.

Parameters	Meaning	Value
imgsz	Input Image Size	640
epochs	The Rounds of Training	300
batch	Batch of Training Data	1
optimizer	Training Optimizer	SGD
Ir0	Initial Learning Rate	0.01
momentum	Momentum Factor	0.937
workers	Worker Thread Count	8

**Table 3:** Confusion Matrix.

Reality	Prediction	
	Positive	Negative
Positive	True Positive (TP)	False Negative (FN)
Negative	False Positive (FP)	True Negative (TN)

**Table 4:** TiN Test Results.

Model	Box (P)	Box (R)	mAP50	Mask (P)	Mask (R)	mAP50
YOLOv8n	0.951	0.641	0.762	0.701	0.475	0.522
SPD0	0.982	0.620	0.809	0.732	0.463	0.542
SPD01	0.975	0.649	0.808	0.749	0.500	0.576
SPD012	0.954	0.633	0.792	0.716	0.478	0.538
SPD0123	0.975	0.641	0.798	0.743	0.491	0.526
SPD01234	0.962	0.644	0.797	0.754	0.506	0.549

**Table 5:** NbC Test Results.

Model	Box (P)	Box (R)	mAP50	Mask (P)	Mask (R)	mAP50
YOLOv8n	0.714	0.494	0.591	0.529	0.373	0.421
SPD0	0.704	0.492	0.581	0.518	0.365	0.396
SPD01	0.737	0.514	0.622	0.557	0.396	0.439
SPD012	0.760	0.483	0.611	0.562	0.360	0.424
SPD0123	0.782	0.512	0.643	0.563	0.372	0.429
SPD01234	0.771	0.501	0.633	0.560	0.365	0.427

**Table 6:** Results of TiN Ablation Experiments.

Model	SPD01	Small	mAP50 (Box)	mAP50 (Mask)
Model01	×	×	0.762	0.522
Model02	√	×	0.808	0.576
Model03	×	√	0.855	0.681
Model04	√	√	0.877	0.730

**Table 7:** Results of NbC Ablation Experiments.

Model	SPD01	Small	mAP50 (Box)	mAP50 (Mask)
Model01	×	×	0.591	0.421
Model02	√	×	0.622	0.439
Model03	×	√	0.745	0.556
Model04	√	√	0.780	0.557

**Table 8:** Image prediction parameter settings.

Parameters	Implication	Value
yolo predict model	Model File	best.pt
source	Image file or path	1.JPG
save	Save the prediction results	True
save_crop	Save the cropped image with the results	True
imgsz	Image size	640
retina_masks	Use high-resolution segmentation mask	True

**Table 9:** Data processing results.

Image	Quantities	Area Fraction	Dispersion	Dispersion-X	Dispersion-Y
1-1#	48	0.72%	2959.4372	1936.8789	2306.4717
1-2#	18	0.21%	1059.4677	836.2208	762.9323
1-3#	60	1.04%	1743.0129	1722.0014	731.2126
1-4#	38	1.08%	1935.6972	808.1959	1863.9304
1-5#	11	0.32%	815.4331	455.6615	773.5230
1-6#	7	0.27%	657.2003	364.0994	622.1773

**Table 10:** Comparison results of different methods.

Image	Statistical Method (Number of Carbonitrides)		
	Manual Statistics	Image-Pro Plus	Modified Model
2-1#	47	101	46
2-2#	52	169	50
2-3#	24	296	25
2-4#	31	222	27
2-5#	57	206	54
2-6#	35	225	38
2-7#	37	203	35
2-8#	22	150	22
2-9#	67	204	62
2-10#	42	159	43

**Table 11:** Comparison of calculation results.

Obj.	P (Model)	R (Model)	P (IPP)	R (IPP)
2-1#	0.978	1.000	0.446	0.957
2-2#	0.960	0.923	0.249	0.808
2-3#	0.960	1.000	0.077	0.958
2-4#	1.000	0.935	0.131	0.935
2-5#	0.981	0.929	0.262	0.947
2-6#	0.922	1.000	0.142	0.914
2-7#	1.000	0.946	0.163	0.892
2-8#	0.857	0.857	0.133	0.909
2-9#	0.984	0.910	0.328	0.910
2-10#	0.953	0.976	0.226	0.857

## Data Availability

The original contributions presented in the study are included in the article, further inquiries can be directed to the corresponding author.
